# Electrochemically Generated ROS Water for Rapid Disinfection and Biofilm Control in Real Waters

**DOI:** 10.3390/microorganisms14030538

**Published:** 2026-02-26

**Authors:** Wending Zhang, Xuerui Ma, Rongchen Jin, Yukun Wang, Long Ren, Shurong Zhang, Lianyu Shan, Kun Cai, Yan Li

**Affiliations:** 1 Department of Pathogen Biology, School of Basic Medicine, Tongji Medical College and State Key Laboratory for Diagnosis and Treatment of Severe Zoonotic Infectious Diseases, Huazhong University of Science and Technology, Wuhan 430030, China; m202575778@hust.edu.cn (W.Z.); xueruima@hust.edu.cn (X.M.); 2 Medical College, Shihezi University, Shihezi 832003, China; 20231006052@stu.shzu.edu.cn; 3 Xiaomi Smart Home Appliances (Wuhan) Co., Ltd., Wuhan 430070, China; wangyukun6@xiaomi.com (Y.W.); renlong1@xiaomi.com (L.R.); zhangshurong@xiaomi.com (S.Z.); shanlianyu@xiaomi.com (L.S.); 4 Institute of Health Inspection and Testing, Provincial Center for Disease Control and Prevention (Hubei CDC), Wuhan 430079, China; 5 Department of Respiratory Medicine, Wuhan Children’s Hospital, Tongji Medical College, Huazhong University of Science and Technology, Wuhan 430014, China; 6 Pediatric Respiratory Disease Laboratory, Institute of Maternal and Child Health, Wuhan Children’s Hospital, Tongji Medical College, Huazhong University of Science and Technology, Wuhan 430014, China; 7 Hubei Provincial Key Laboratory of Pediatric Genetic Metabolic and Endocrine Rare Diseases, Wuhan 430030, China

**Keywords:** electrochemical disinfection, boron-doped diamond, reactive oxygen species, real water matrices, biofilm disruption

## Abstract

The intensifying global challenges of water scarcity and widespread microbial contamination underscore the urgent need for the development of efficient, chemical-free disinfection technologies. Here, we developed a compact boron-doped diamond (BDD)-based electrochemical water treatment system that generates reactive oxygen species (ROS) in situ and evaluated its antimicrobial performance using ROS-on/off controls. Bactericidal efficacy was assessed against representative Gram-negative *Escherichia coli* (*E. coli*), Gram-positive *Staphylococcus aureus* (*S. aureus*), and *Pseudomonas aeruginosa* (*P. aeruginosa*), a clinically relevant Gram-negative pathogen with biofilm-forming and stress-resistant properties. Under ROS-on operation, viable counts were reduced from ~10^6^ CFU/mL to near the detection limit, corresponding to 5–6 log10 reductions across all tested species, whereas ROS-off treatment showed negligible effects. The system retained strong disinfection activity in complex real water matrices, including hand-washing water, laboratory wastewater, and pond wastewater. ROS-treated water also disrupted pre-formed mono-species biofilms in a time-dependent manner, as assessed by crystal violet staining and semi-quantitative biomass analysis. A preliminary mouse exposure assessment did not reveal obvious histopathological abnormalities or hematological changes under the tested conditions. These results demonstrate that BDD-enabled electrochemical ROS water provides a rapid, reagent-free approach for bacterial inactivation and biofilm control, with potential applicability across diverse water-related settings, while acknowledging that further studies on complex natural microbial communities are warranted.

## 1. Introduction

The escalating global challenge of water scarcity is compounded by the pervasive threat of microbial contamination, which remains a leading cause of morbidity and mortality worldwide, exacerbated by rapid urbanization and the increasing frequency of extreme weather events [1,2]. Conventional sterilization and disinfection methods, while historically effective, are increasingly constrained by contemporary technical and environmental requirements. Thermal treatment is energy-intensive and unsuitable for heat-sensitive environments; chemical disinfection, particularly chlorination, often generates potentially carcinogenic disinfection byproducts and requires complex supply chains for reagent storage; ultraviolet irradiation suffers from low penetration in turbid waters and the “shadowing effect” created by suspended solids [3,4,5]. Consequently, there is an urgent demand for next-generation disinfection technologies that are reagent-free, modular, and robust across diverse operational settings [6].

The complexity of real-world water environments presents a formidable barrier to efficient disinfection. In most aquatic systems and on wet surfaces, bacteria do not exist solely in a planktonic state but predominantly organize into biofilms—complex, self-produced matrices of extracellular polymeric substances [7,8,9]. These biofilms provide a protective microenvironment, rendering the encased bacteria up to 1000 times more resistant to traditional antimicrobial agents than their free-floating counterparts [10,11]. This biofilm-associated tolerance leads to persistent contamination and system failure in water distribution networks. Furthermore, real-world water matrices contain high levels of organic matter, inorganic ions, and turbidity, all of which competitively consume disinfectants or reduce the free path of active species, significantly weakening disinfection efficacy [12,13,14]. While not the primary focus, the underlying pressure of antimicrobial resistance further underscores the need for non-specific, rapid-acting inactivation mechanisms [15].

To address these challenges, innovative strategies based on reactive oxygen species (ROS) have emerged as promising candidates. Technologies such as non-thermal plasma, photocatalysis, and electrochemical advanced oxidation processes leverage the potent, non-specific oxidative power of species like hydroxyl radicals (⋅OH), ozone (O_3_), to achieve rapid microbial inactivation [16,17,18,19]. Among these, electrochemical systems utilizing BDD anodes are widely regarded as a benchmark for reagent-free water remediation [20]. Compared with conventional anodes, BDD is less prone to corrosion and can sustain high anodic polarization, making it suitable for in situ oxidant generation. BDD’s exceptionally wide electrochemical potential window and low capacitive current allow for the efficient generation of high-concentration ROS directly from water molecules without the need for additional chemical inputs [21,22]. However, a critical gap remains between laboratory-scale material research and practical application. Much of the existing literature is performance-centric, focusing on idealized conditions while lacking evidence under dynamic recirculating operations, multi-matrix real water environments, and, crucially, a comprehensive evaluation of the systemic toxicity and biocompatibility of ROS-treated water [23,24].

**Figure 1:**
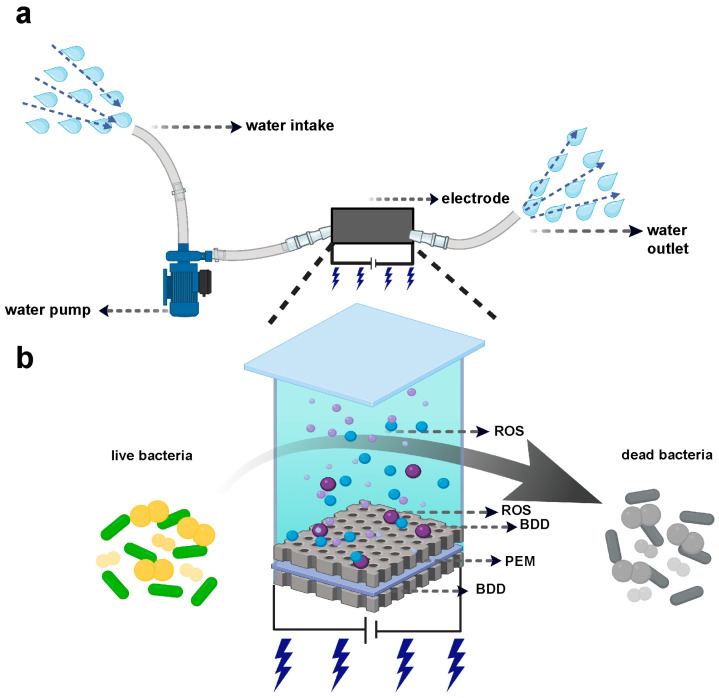
Schematic illustration of the BDD-based electrochemical water treatment system and its ROS-mediated disinfection mechanism. (**a**) Diagram of the continuous-flow electrochemical water treatment setup. Influent water is pumped into the electrochemical reactor equipped with BDD electrodes and collected at the outlet after treatment. (**b**) Schematic of the electrochemical disinfection mechanism. Upon application of an external potential, the BDD electrodes generate ROS in situ at the electrode–electrolyte interface, with the PEM facilitating ion transport. The generated ROS diffuse into the bulk solution and induce oxidative damage to bacterial cells, resulting in bacterial inactivation. Panel (**a**) was created in BioRender. Y.L. (2026) https://BioRender.com/91qcedo (accessed on 1 January 2026). Panel (**b**) was created in BioRender. Y.L. (2026) https://BioRender.com/j352hp3 (accessed on 1 January 2026).

In this study, we evaluate a compact BDD-based electrochemical system for reagent-free water disinfection and biofilm control. As schematically illustrated in Figure 1, the system operates in a continuous-flow configuration, in which influent water is pumped through an electrochemical reactor equipped with BDD electrodes and collected after treatment. Within the reactor, the BDD electrodes, separated by a proton exchange membrane (PEM), enable in situ generation of reactive oxidants under an applied electric field. We perform electrochemical characterization—including linear sweep voltammetry (LSV) and electrochemical impedance spectroscopy (EIS)—to establish the anodic behavior and interfacial properties of the BDD electrode relevant to oxidant generation. We then assess antibacterial performance against representative Gram-negative *Escherichia coli* (*E. coli*), Gram-positive *Staphylococcus aureus* (*S. aureus*) and *Pseudomonas aeruginosa* (*P. aeruginosa*), a clinically relevant Gram-negative pathogen with biofilm-forming and stress-resistant properties. Extending beyond lab-scale benchmarks, we evaluate the system’s efficacy in diverse real water matrices, including laboratory wastewater and pond wastewater, and under dynamic recirculating conditions. Importantly, we also demonstrate the time-dependent disruption and removal of pre-formed mono-species bacterial biofilms. Finally, to address the safety gap, we conducted a preliminary in vivo toxicity study in a murine model. Our results show that exposure to ROS-treated water via topical and oral routes did not induce detectable tissue damage or hematological abnormalities. Together, these findings demonstrate that BDD-based electrochemical ROS treatment is a promising reagent-free approach for water disinfection and biofilm control, with potential applicability across diverse water scenarios, while further studies are required to evaluate its efficacy against multi-species microbial communities in natural environments.

## 2. Materials and Methods

### 2.1. Electrochemical Measurements

The working electrodes were provided by Xiaomi Smart Home Appliances (Wuhan) Co., Ltd., Wuhan, China. Unless otherwise specified, the electrodes were used as received without further modification. Electrochemical measurements were carried out using a CHI 660E electrochemical workstation (Shanghai Chenhua Instrument Co., Ltd., Shanghai, China) with a conventional three-electrode configuration. The BDD electrode served as the working electrode, a platinum foil (Pt foil) was used as the counter electrode, and an Ag/AgCl electrode was employed as the reference electrode. All electrochemical measurements were conducted at room temperature. The potentials measured against the Ag/AgCl reference electrode were converted to values relative to the reversible hydrogen electrode (RHE) according to the following equation:

$$E_{\mathrm{RHE}}{\;=\;E}_{\mathrm{Ag}/\mathrm{AgCl}}\;+\;0.197\;+\;0.059\;\times \;\mathrm{pH}$$

E_RHE_ represents the potential versus RHE, E_Ag/AgCl_ is the measured potential versus the Ag/AgCl reference electrode, and pH is that of the electrolyte. EIS measurements were performed in the potential range of 1.6–2.8 V (vs. RHE), with an AC perturbation amplitude of 5 mV and a frequency range from 100 kHz to 0.1 Hz.

### 2.2. X-Ray Diffraction (XRD) Characterization

The phase composition was analyzed by XRD. XRD measurements were performed using a Rigaku Ultima III X-ray diffractometer (Rigaku Corporation, Tokyo, Japan). Cu Kα radiation was employed as the X-ray source, with a wavelength of λ = 0.154 nm. The diffraction patterns were collected over a 2θ range of 10–80°, with a step size of 0.02° and a scanning rate of 2–10°/min. The instrument was operated at an accelerating voltage of 40 kV and a tube current of 40 mA.

### 2.3. Morphological Characterization

The surface morphology of the BDD coatings was characterized by field emission scanning electron microscopy (FE-SEM; Nova NanoSEM 230, FEI, Hillsboro, OR, USA). Prior to observation, the electrodes were ultrasonically cleaned in ethanol for 15 min to remove surface contaminants and subsequently dried under ambient conditions.

### 2.4. Bacterial Strains and Culture Conditions

*S. aureus* (ATCC 25923), *P. aeruginosa* (ATCC 27853) and *E. coli* (ATCC 25922) were used as representative bacterial models. The bacterial strains were streaked onto Luria–Bertani (LB) agar plates using the three-zone streaking method and incubated inverted at 37 °C overnight. Single colonies were then picked from the agar plates and inoculated into 5 mL of LB broth, followed by incubation at 37 °C with shaking at 200 rpm overnight. On the following day, the optical density of the bacterial suspensions was measured at 600 nm (OD_600_) using a microplate reader. The overnight cultures were subsequently diluted 1:100 (*v*/*v*) into fresh LB medium and incubated at 37 °C with shaking at 200 rpm for approximately 3 h to obtain bacteria in the logarithmic growth phase. These freshly prepared bacterial suspensions were immediately used for subsequent experiments.

### 2.5. Disinfection Performance

All instruments and materials used in the experiments were sterilized by autoclaving prior to use. Bacterial cultures in the logarithmic growth phase were harvested by centrifugation at 2380× *g* for 10 min. After removal of the supernatant, the bacterial pellets were resuspended in 250 mL of sterile 0.9% (*w*/*v*) saline solution. The initial bacterial concentration for the experiments was adjusted to 1 × 10^6^ CFU/mL using a tenfold serial dilution method with sterile saline. The disinfection device was thoroughly sterilized with 75% ethanol before each experiment. Under the single-pass operating mode, the inlet of the device was immersed in the pre-prepared bacterial suspension at the beginning of the experiment, and the treated effluent was collected in a separate sterile conical flask at the outlet. According to the experimental design, electrochemical polarization was either applied (ROS-on) or not applied (ROS-off). In the recirculation mode, both the inlet and outlet of the device were placed in the same container, allowing the bacterial suspension to circulate continuously through the device. At predetermined time points, treated samples were collected and subjected to subsequent analyses.

### 2.6. Colony-Forming Unit (CFU) Enumeration

After treatment, the bacterial suspensions were subjected to tenfold serial dilutions with sterile saline, and appropriate dilution factors were selected according to the experimental conditions prior to plating. An aliquot of 100 μL from each diluted suspension was evenly spread onto LB agar plates and incubated at 37 °C overnight. On the following day, the agar plates were photographed, and the number of colonies was quantified using ImageJ (version 1.54g). Bacterial viability was expressed as CFU.

$$\mathrm{CFU}/\mathrm{mL}=\frac{\mathrm{colony}\;\mathrm{count}\;\times \;\mathrm{dilution}\;\mathrm{ratio}}{\mathrm{sample}\;\mathrm{volume}}$$

The antibacterial (inhibition) efficiency was calculated according to the following equation:

$$\mathrm{Antibacterial}\;\mathrm{efficiency}\;=\;\frac{B_{\mathrm{before}}-B_{\mathrm{after}}}{B_{\mathrm{before}}}\;\times \;100\%$$

### 2.7. Live/Dead Bacterial Staining

Live/dead bacterial staining was performed using a commercially available Live/Dead Bacterial Staining Kit (Solarbio, Beijing, China; catalog no. EX3000). The kit contains two fluorescent nucleic acid dyes: NucGreen, a green fluorescent stain that penetrates both live and dead bacteria, and EthD-III, a red fluorescent dye that selectively stains bacteria with compromised cell membranes. When used in combination, NucGreen and EthD-III enable discrimination between live and dead bacterial cells. Bacteria with intact cell membranes exhibit green fluorescence, while bacteria with damaged membranes display red fluorescence, with overlapping signals appearing green and red in the corresponding fluorescence channels. Following the staining procedure, bacterial viability and membrane integrity were visualized using a laser scanning confocal microscope (Olympus FV3000, Tokyo, Japan).

### 2.8. Biofilm Removal Assay

Biofilms were formed in sterile 24-well plates with 1 mL LB medium per well and an initial bacterial concentration of 1 × 10^7^ CFU/mL, incubated statically at 37 °C for 24–48 h. Non-adherent bacteria were removed by gentle PBS washes, and the biofilms were air-dried for 1 h. Plates were treated with ROS water or controls for designated times, followed by PBS washes. Biofilms were stained with 0.1% (*w*/*v*) crystal violet for 45 min in the dark, washed with PBS, and air-dried. For quantification, 1 mL of 30% (*v*/*v*) acetic acid was added per well to solubilize the dye. Aliquots (200 μL) were transferred to a 96-well plate, and absorbance at 562 nm was measured. Biofilm removal percentage was calculated relative to control groups. All experiments were performed in triplicate at minimum, and the results are presented as mean ± standard error of the mean (mean ± SEM). The percentage of biofilm removal inhibition was calculated by comparing the average OD_562_ values of the treated groups with those of the control group according to the following equation:

$$\mathrm{Biofilm}\;\mathrm{destruction}\;(\%)\;=\;\left(1-\frac{{\mathrm{OD}}_{\mathrm{treated}}-{\mathrm{OD}}_{\mathrm{blank}}}{{\mathrm{OD}}_{\mathrm{control}}-{\mathrm{OD}}_{\mathrm{blank}}}\right)\times 100\%$$

### 2.9. Mouse Model

This study was approved by the Institutional Animal Care and Use Committee (IACUC) of Huazhong University of Science and Technology (IACUC Number: 5002). All animal experiments were conducted in accordance with the National Institutes of Health (NIH) Guide for the Care and Use of Laboratory Animals. Female BALB/c mice (8 weeks old, average body weight 20 ± 2 g) were housed under specific-pathogen-free (SPF) conditions with a 12 h light/dark cycle and had ad libitum access to sterilized feed and water. Mice were acclimated for 7 days prior to experimentation. Animals were randomly assigned to treatment or control groups for dermal and oral exposure experiments (*n* = 4 per group) using a random number table. Mice were labeled for subsequent procedures and observations.

For dermal exposure, four mice received topical application of 0.2 mL ROS-treated water to the ear skin, while control mice received the same volume of untreated water under identical conditions. For oral exposure, four mice were provided with ROS-treated water ad libitum for 24 h, whereas another four mice receiving autoclaved water served as the control group.

### 2.10. Hematoxylin and Eosin (H&E) Staining

Following anesthesia and euthanasia, mouse tissues were harvested and fixed in 4% paraformaldehyde (PFA, pH 7.4). The specimens were embedded in paraffin and sectioned into 5 μm thick slices. For histological analysis, sections were deparaffinized, rehydrated, and stained with hematoxylin for 5 min. After rinsing in tap water, the sections were differentiated in 1% acid alcohol (1% HCl in 70% ethanol) for 30 s and subsequently counterstained with 0.5% aqueous eosin for 2 min. Finally, the sections were dehydrated through a graded ethanol series, cleared in xylene, and mounted with neutral resin under a glass coverslip. Care was taken to avoid air bubbles during mounting.

The stained sections were examined under an optical microscope to observe and record the number and distribution of inflammatory cells, as well as tissue proliferation. For systemic analysis, blood samples were obtained from the remaining mice under anesthesia. Following euthanasia, the heart, liver, and kidney tissues were excised, fixed, and processed for H&E staining according to the aforementioned protocol.

### 2.11. Complete Blood Count (CBC) Analysis

Whole blood samples containing EDTA-K_2_ anticoagulant were collected and analyzed using an automated hematology analyzer (BC-2800Vet). The following parameters were measured: White blood cell (WBC) system: total WBC count, and differential counts and percentages of granulocytes (GRAN), lymphocytes (LYM), and monocytes (MON). Red blood cell (RBC) system: RBC count, hematocrit (HCT), mean corpuscular volume (MCV), red cell distribution width (RDW), hemoglobin (HGB), mean corpuscular hemoglobin (MCH), and mean corpuscular hemoglobin concentration (MCHC). Platelet system: platelet count (PLT), mean platelet volume (MPV), platelet distribution width (PDW), and plateletcrit (PCT). Quality control procedures were conducted in accordance with CLSI H20-A2 standards. The analyzer was calibrated daily using the accompanying QC materials at three concentration levels.

### 2.12. Statistical Analysis

Data are expressed as mean ± SEM. Differences between groups were analyzed by Student’s t-test or one-way ANOVA with Tukey’s post hoc test (GraphPad Prism 10.1.2). Significance was set at *p* < 0.05.

## 3. Results

### 3.1. Electrochemical and Structural Characterization of the BDD Electrode

**Figure 2:**
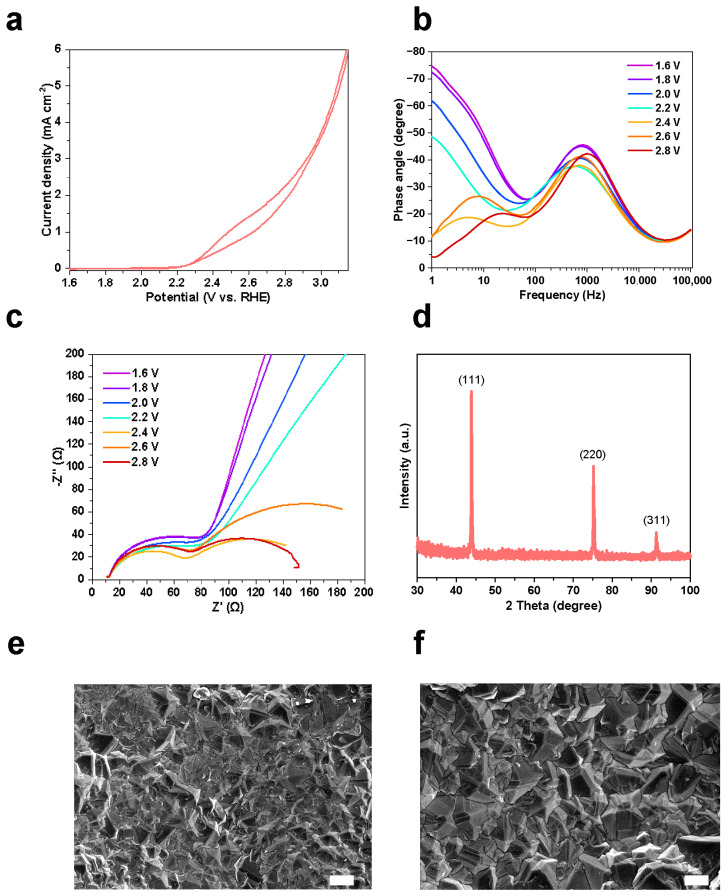
Electrochemical and structural characterization of the BDD electrode. (**a**) LSV polarization curve of the BDD electrode; (**b**) Bode phase plots obtained from EIS measurements under different applied potentials; (**c**) Corresponding Nyquist plots, displaying two distinct semicircles associated with different interfacial charge-transfer processes; (**d**) XRD pattern of the electrode coating; and (**e,f**) SEM images of the BDD surface at different magnifications, with scale bars of 20 μm (**e**) and 10 μm (**f**).

To establish the electrochemical properties of the BDD electrode and provide a basis for subsequent disinfection studies, its electrochemical behavior and structural characteristics were first examined. The electrochemical behavior of the BDD electrode was evaluated by LSV (Figure 2a). A distinct anodic current onset was observed at approximately 2.4 V (vs. RHE), indicating the initiation of anodic reactions. Upon increasing the applied potential to 3.0 V, the current density increased sharply to 3.6 mA cm^−2^, reflecting a sustained anodic current response at high potentials. EIS was employed to further elucidate the interfacial charge-transfer kinetics (Figure 2b,c). The Nyquist plots exhibit two distinguishable impedance features over the investigated potential range, corresponding to two kinetically distinguishable interfacial processes. The high-frequency semicircle is associated with charge transfer at the BDD-substrate interface, whereas the low-frequency semicircle corresponds to electron transfer at the electrolyte–electrode interface. The radius of the high-frequency semicircle remains essentially unchanged with increasing applied potential, indicating minimal variation in the electronic response of the BDD film. In contrast, the radius of the low-frequency semicircle decreases markedly when the applied potential exceeds the onset potential of 2.4 V, consistent with enhanced interfacial charge transfer kinetics. This behavior suggests the activation of additional faradaic processes at elevated anodic potentials. The Bode phase plots further support this trend (Figure 2b). With increasing applied potential beyond the onset value, the characteristic low-frequency phase peak shifts from approximately 1 Hz to 10 Hz, accompanied by a reduction in peak intensity. Such a frequency shift is consistent with accelerated electron transfer processes observed in the Nyquist analysis.

The crystalline structure of the electrode coating was characterized by XRD (Figure 2d). The diffraction pattern exhibits characteristic reflections corresponding to the (111), (220), and (311) planes, confirming the formation of a pure diamond phase without detectable graphitic or amorphous carbon. Notably, the dominance of the (111) peak indicates a strong preferred orientation of the BDD electrode. This structural alignment is advantageous, as it favors the exposure of abundant boron active sites. Scanning electron microscopy (SEM) images reveal a continuous and densely packed polycrystalline morphology of the BDD coating (Figure 2e,f). Well-defined faceted diamond grains are uniformly distributed across the surface, indicating a continuous polycrystalline film morphology. Together, these results confirm the electrochemical stability and structural integrity of the BDD electrode under anodic polarization, providing a reliable foundation for subsequent disinfection performance evaluation.

### 3.2. Bactericidal Activity of the BDD-Based Electrochemical System

Building on the electrochemical characteristics of the BDD electrode, we next evaluated the bactericidal efficacy of the BDD-based electrochemical water treatment system against representative Gram-negative (*E. coli*, *P. aeruginosa*) and Gram-positive (*S. aureus*) bacteria. Experimental groups were defined by the activation of the ROS module: ROS(-) indicates that ROS generation was disabled, whereas ROS(+) denotes continuous ROS generation during treatment.

**Figure 3:**
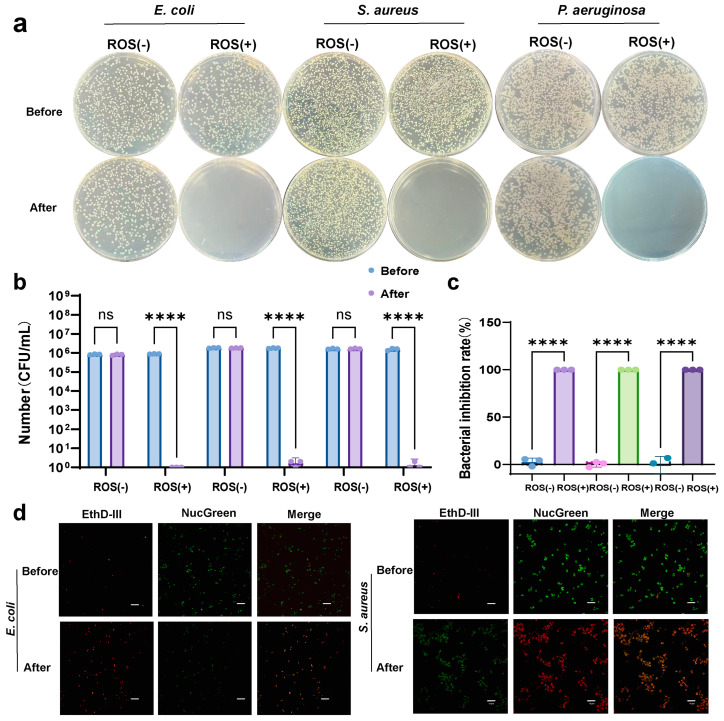
ROS-dependent bactericidal activity of the BDD-based electrochemical system. (**a**) Representative agar plate images of *E. coli*, *S. aureus*, and *P. aeruginosa* before and after treatment under ROS(-) and ROS(+) conditions using the BDD electrode device; (**b**) Quantitative analysis of bacterial survival based on CFU enumeration; (**c**) Calculated bacterial inhibition rates under different treatment conditions; and (**d**) Live/dead fluorescence staining (EthD-III/NucGreen) images showing bacterial membrane integrity and viability after treatment. Scale bar, 10 μm. For (**b**,**c**): n  =  3 independent measurements; data are presented as the means  ±  SEM. **** *p* < 0.0001 versus before treatment.

Representative agar plate images revealed that colony formation for all three species remained largely unchanged under ROS(-) conditions (Figure 3a). In contrast, ROS(+) treatment nearly eradicated observable colonies, indicating potent bactericidal activity. Quantitative CFU analysis further supported these observations (Figure 3b). Under ROS(-) conditions, bacterial loads remained at approximately 10^6^ CFU/mL for each species, whereas ROS(+) treatment reduced CFU counts to near the detection limit, corresponding to 5–6 log10 reductions. Accordingly, calculated inhibition rates approached 100% under ROS(+) conditions, while remaining minimal under ROS(-) conditions (Figure 3c). Importantly, *P. aeruginosa* exhibited similar susceptibility to ROS-mediated inactivation as *E. coli* and *S. aureus* within the scope of the strains tested, suggesting effectiveness across these representative Gram-negative and Gram-positive bacteria.

Live/dead fluorescence staining (EthD-III/NucGreen) further supported bacterial inactivation at the cellular level. Under ROS(-) conditions, bacteria predominantly exhibited green fluorescence, indicating intact membranes. Overall, these results demonstrate that the BDD-based system achieves rapid and reproducible bactericidal activity against the tested bacterial strains, consistent with ROS-induced membrane damage. Collectively, plate assay images, CFU enumeration, inhibition-rate analysis, and fluorescence staining consistently support ROS-dependent bacterial inactivation, providing a foundation for further evaluation under more complex microbial communities and water conditions.

### 3.3. Disinfection Performance in Real-Water Matrices and Biofilm Removal

**Figure 4:**
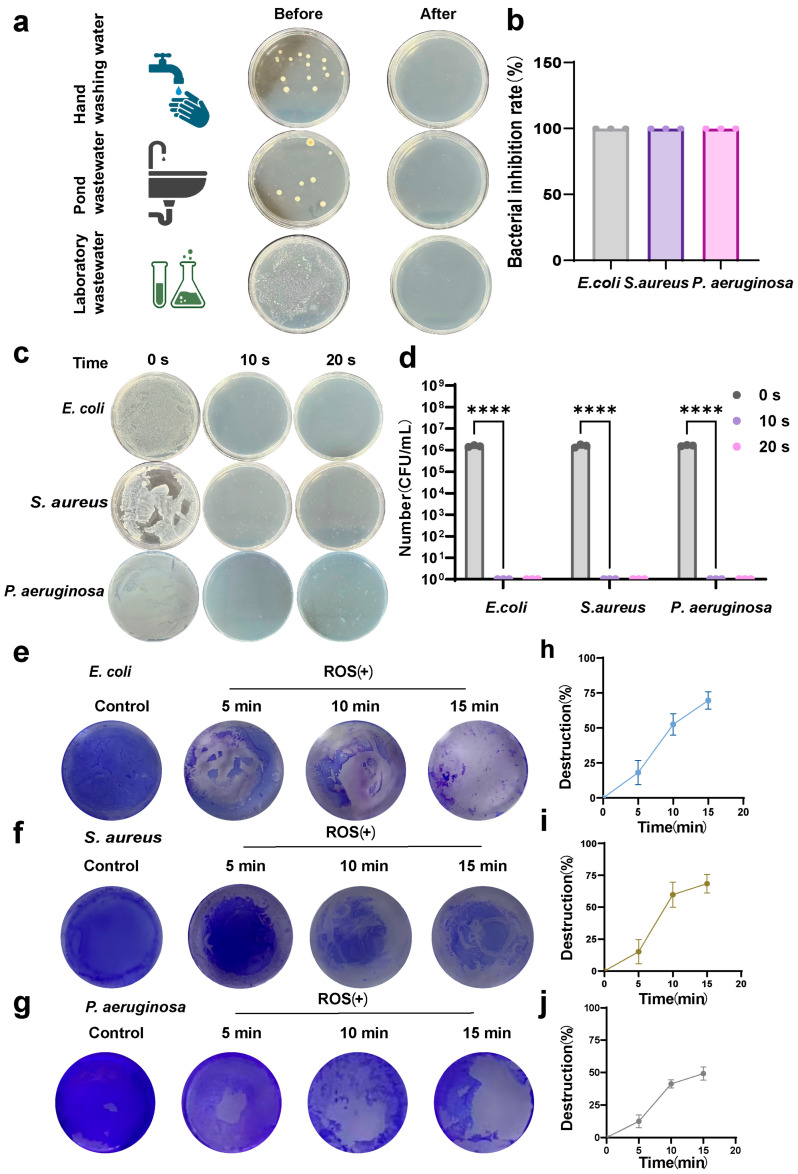
Disinfection performance of the BDD-based electrochemical system in real water matrices and biofilm removal. (**a**) Representative plating images showing colony formation in hand-washing water, laboratory wastewater, and pond wastewater before and after disinfection with the BDD-based electrochemical water treatment system; (**b**) Corresponding bactericidal efficiencies calculated in a 250 mL recirculating system; (**c**) Time-dependent reduction in *E. coli*, *S. aureus*, and *P. aeruginosa* colony counts in a 250 mL recirculating system; (**d**) Quantification of surviving bacteria based on CFU counts after different treatment durations; (**e**–**g**) Disruption of *E. coli* (**e**), *S. aureus* (**f**), and *P. aeruginosa* (**g**) biofilms visualized by crystal violet staining; and (**h**–**j**) Semi-quantitative analysis of residual biofilm biomass for *E. coli* (**h**), *S. aureus* (**i**), and *P. aeruginosa* (**j**). For (**b**,**d**,**h**–**j**): n  =  3 independent measurements; data are presented as the means ± SEM. **** *p* < 0.0001 versus before treatment. Panel (**a**) was created in BioRender. Y.L. (2026) https://BioRender.com/xwhlizi (accessed on 1 January 2026).

To evaluate the disinfection performance of the BDD-based electrochemical water treatment system under realistic conditions, various real water samples, including hand-washing water, laboratory wastewater, and pond wastewater, were treated and analyzed (Figure 4a). Plate-counting results showed substantial colony growth in untreated samples, whereas treatment with the BDD system led to a near-complete elimination of visible colonies, indicating effective bactericidal activity in complex water matrices. The rapid disinfection performance of the system was further examined using a 250 mL recirculating setup to simulate dynamic operational conditions (Figure 4b–d). Representative planktonic bacteria, including *E. coli*, *S. aureus*, and *P. aeruginosa*, displayed a pronounced, time-dependent decrease in colony numbers. After 20 s of treatment, bacterial loads were reduced by several orders of magnitude relative to untreated controls, with statistically significant differences. Corresponding bactericidal efficiencies approached 100% for the tested strains, demonstrating rapid and effective antibacterial activity under these conditions. It should be noted that these rapid disinfection results are limited to the selected laboratory strains tested here.

Beyond planktonic bacteria, the effect of ROS generated by the BDD system on pre-established single-species biofilms was investigated. Crystal violet staining revealed time-dependent disruption of biofilm structures for *E. coli*, *S. aureus*, and *P. aeruginosa* (Figure 4e–j). Untreated biofilms were dense and compact, whereas ROS treatment (5, 10, and 15 min) induced progressive biofilm disintegration and detachment, resulting in substantial reduction in surface coverage. Semi-quantitative analysis based on crystal violet absorbance measurements confirmed a continuous decrease in residual biofilm biomass for all three species with increasing treatment duration, indicating effective biofilm removal. Together, these results indicate that the BDD-based electrochemical water treatment system can efficiently eliminate planktonic cells and disrupt single-species biofilms in real water samples. However, the current findings are restricted to monospecies biofilm models, and the efficacy of the system against multispecies or naturally occurring microbial communities remains to be evaluated.

### 3.4. Preliminary Toxicity Evaluation of ROS Water in Mice

**Figure 5:**
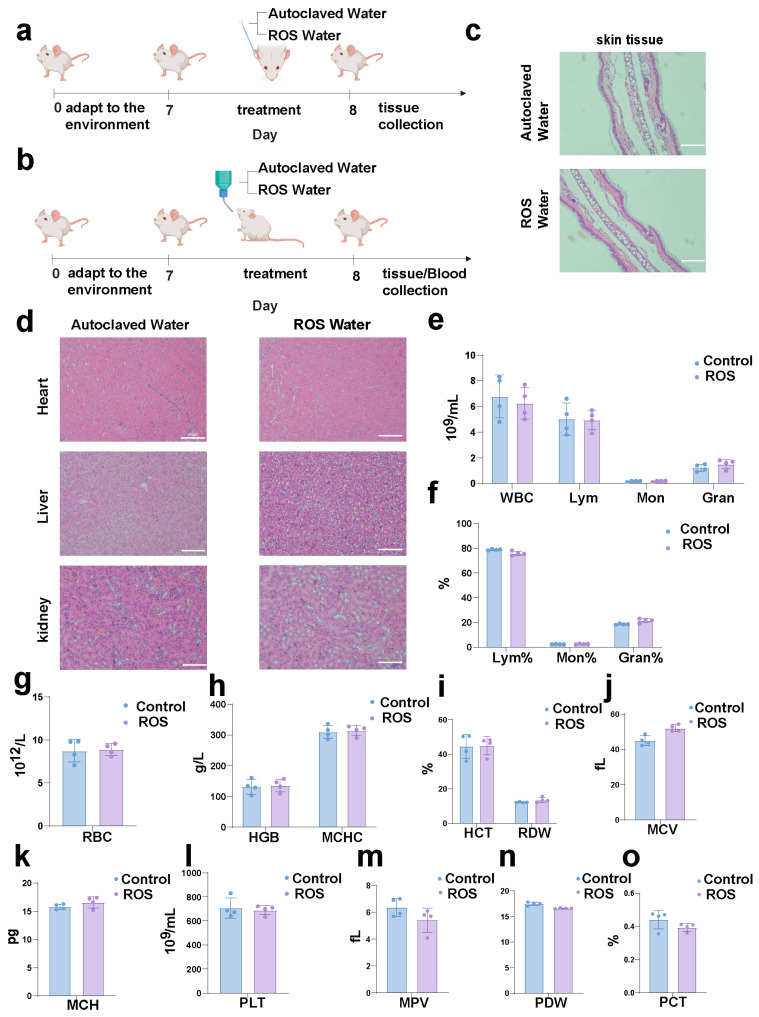
Preliminary toxicity study of ROS water treatment in mice. (**a**,**b**) Schematic illustration of the experimental design and treatment timeline for evaluating the short term toxicity of ROS water exposure. (**c**) Representative H&E-stained images of skin tissues collected from mice treated with autoclaved water (control) or ROS water. Scale bar: 10 μm. (**d**) Representative H&E-stained sections of heart, liver, and kidney of mice given autoclaved water (control) or ROS water ad libitum. Scale bar: 10 μm. (**e**) Total white blood cell (WBC) counts in peripheral blood following treatment. (**f**) Differential leukocyte percentages, including lymphocytes (Lym), monocytes (Mon), and granulocytes (Gran). (**g**–**k**) Red blood cell-related hematological parameters, including red blood cell (RBC) count, hemoglobin (HGB), mean corpuscular hemoglobin concentration (MCHC), hematocrit (HCT), red cell distribution width (RDW), mean corpuscular volume (MCV), mean corpuscular hemoglobin (MCH). (**l**–**o**) Platelet-related parameters, including platelet count (PLT), mean platelet volume (MPV), platelet distribution width (PDW), and plateletcrit (PCT). Data in (**e**–**o**) are shown as mean  ±  SEM, with n = 4 biological replicates. Panel (**a**) was created in BioRender. Y.L. (2026) https://BioRender.com/sacm8l0 (accessed on 1 January 2026). Panel (**b**) was created in BioRender. Y.L. (2026) https://BioRender.com/q2uxrgp (accessed on 1 January 2026).

To complement these efficacy results, we next conducted a preliminary in vivo safety evaluation to assess potential acute adverse effects of ROS water exposure via dermal (topical) and oral routes. Following a 7-day acclimatization period, mice were exposed to ROS water or autoclaved water (control), and tissues/blood were collected on day 8 (Figure 5a,b). Throughout the exposure period, mice in both groups maintained normal activity, feeding, and grooming habits, with no observable signs of distress, lethargy, or abnormal behavior. No significant differences in general health status or body condition were noted between the ROS-treated and control groups. Histological analysis of skin tissues showed intact epidermal and dermal layers in both groups, with no detectable signs of epithelial damage, inflammatory cell infiltration, or tissue edema following topical exposure of the ear skin to ROS water (Figure 5c). Consistently, H&E staining of major organs, including the heart, liver, and kidney, collected from mice provided with ROS water ad libitum showed well-preserved tissue architecture, which was comparable to that of the control group (Figure 5d). No obvious pathological alterations, such as necrosis, cellular degeneration, or inflammatory lesions, were observed.

To further evaluate the potential systemic toxicity induced by oral intake of ROS water, hematological parameters in peripheral blood were comprehensively analyzed. Total white blood cell (WBC) counts and differential leukocyte populations, including lymphocytes (Lym), monocytes (Mon), and granulocytes (Gran), showed no significant differences between ROS-treated and control groups (Figure 5e,f), suggesting that ROS water exposure did not induce detectable immune dysregulation. Red blood cell-related indices, including red blood cell (RBC) count, hemoglobin (HGB), mean corpuscular hemoglobin concentration (MCHC), hematocrit (HCT), red cell distribution width (RDW), mean corpuscular volume (MCV), and mean corpuscular hemoglobin (MCH), remained comparable between the two groups (Figure 5g–k). In addition, platelet-associated parameters, including platelet count (PLT), mean platelet volume (MPV), platelet distribution width (PDW), and plateletcrit (PCT), were not significantly altered following ROS water treatment (Figure 5l–o). Overall, within the tested exposure conditions and duration, no obvious tissue damage or hematological abnormalities were observed in mice treated with ROS water, indicating no detectable acute toxicity in this preliminary evaluation.

## 4. Discussion

This study evaluates a compact BDD-based electrochemical system for reagent-free disinfection and biofilm control. Anodic polarization of the BDD electrode enables rapid, ROS-dependent inactivation of representative Gram-negative and Gram-positive bacteria, effective disruption of pre-established biofilms, and maintained performance in real water matrices. Preliminary murine exposure tests indicate no detectable acute toxicity via dermal or oral routes. In the following, we discuss how the electrochemical properties of BDD support ROS generation, why the system remains effective in complex matrices and biofilm settings, and the implications and limitations of the initial safety results.

The BDD electrode exhibits a wide anodic window and high oxygen evolution overpotential (OEP ≈ 2.2–2.8 V vs. RHE), enabling efficient ROS generation while limiting water decomposition and electrode degradation [25]. The sharp current rise to ~3.6 mA at 3.0 V underscores a potent oxidation capacity, driven by the formation of reactive intermediates at both the electrode interface and in the bulk solution. EIS analysis further confirms the BDD’s electronic stability; the potential-independent high-frequency semicircle indicates that intrinsic charge transport remains robust under anodic polarization—a resilience attributed to the sp^3^ carbon lattice and stable boron doping [26]. Beyond the ~2.4 V onset, the significant contraction of the low-frequency semicircle radius confirms accelerated electron transfer, signaling the activation of Faradaic pathways for ROS production. These electro-generated species, primarily ·OH and O_3_, serve as the main oxidants; ·OH is particularly lethal to bacteria in chloride-free media, while O_3_ provides supplementary oxidative stress at elevated potentials [27]. XRD and SEM revealed dense, pure polycrystalline diamond, providing electroactive surface area and conductive pathways for sustained ROS generation [26].

The BDD-based electrochemical water treatment system showed broad-spectrum efficacy against both Gram-negative *E. coli* and Gram-positive *S. aureus*, with ROS(+) conditions yielding > 5–6 log reductions in CFU and near-complete inactivation, while ROS(-) treatment had negligible impact. These findings are consistent with prior electrochemical disinfection studies showing that ROS generated in situ at BDD anodes can achieve multi-log inactivation of waterborne pathogens under appropriate operating conditions [28]. The live/dead staining further supported bacterial inactivation at the cellular level, revealing extensive membrane compromise consistent with oxidative damage. Such membrane disruption has been widely reported as a primary pathway for ROS-mediated microbial inactivation [27]. Importantly, the system maintained bactericidal activity in complex real water matrices (hand-washing water, laboratory wastewater, and pond wastewater) and under dynamic recirculation conditions, with rapid time-dependent CFU reductions approaching detection limits. This observation suggests electrochemically generated oxidants remain effective in the presence of organic matter and scavengers typically found in environmental waters. The observed biofilm removal further highlights the capability of the ROS-rich oxidative environment to disrupt resistant microbial communities. Crystal violet staining and quantitative biomass reduction with treatment time indicate progressive disintegration of biofilm matrices. The preliminary in vivo toxicity results provide initial evidence that exposure to ROS water at the tested doses did not induce acute adverse physiological impacts in murine models. These findings align with the environmental compatibility profile often attributed to electrochemical advanced oxidation technologies when operated within optimized potential windows that minimize formation of harmful disinfection byproducts. This apparent “selective toxicity” may be partly attributable to endogenous antioxidant defenses in mammals (e.g., catalase and glutathione peroxidase), which can scavenge low-level reactive species, whereas such protective capacity is absent or less effective in unicellular microbes [14].

From a techno-economic perspective, conventional chlorination remains widely implemented due to low direct operating costs but requires continuous chemical dosing and management of disinfection by-products [29]. Ultraviolet irradiation provides reagent-free disinfection yet is limited by photon attenuation and recurring lamp replacement [30], whereas ozonation typically incurs higher capital and energy demand associated with on-site oxidant generation [31]. In contrast, electrochemical oxidation using boron-doped diamond electrodes operates through on-demand oxidant production without chemical storage and benefits from the exceptional durability of diamond anodes, which supports long-term cost amortization and stable performance [22,32]. Although the instantaneous energy cost may exceed that of chlorination in large centralized systems, the absence of chemical logistics, reduced secondary pollution, and modular scalability indicate that BDD-based electrochemical disinfection represents an economically competitive and operationally resilient alternative, particularly in decentralized or resource-limited water treatment scenarios.

In summary, the BDD-based electrochemical system provides an efficient, chemical-free platform for water disinfection and biofilm eradication. Its high anodic stability and oxygen evolution overpotential enable on-demand generation of reactive oxidants, achieving multi-log inactivation of the tested Gram-negative and Gram-positive strains and effective dismantling of mature biofilms. Efficacy is maintained across complex water matrices, demonstrating resilience against radical scavengers under practical conditions. Preliminary in vivo tests indicate no acute toxicity, tissue damage, or hematological abnormalities. These findings substantiate BDD-based electrochemical oxidation as a sustainable and scalable approach for point-of-use water purification and broad waterborne pathogen control, advancing next-generation green disinfection strategies.

## Data Availability

The original contributions presented in this study are included in the article. Further inquiries can be directed to the corresponding authors.

## Abbreviations

The following abbreviations are used in this manuscript:

ANOVA	analysis of variance
ATCC	American Type Culture Collection
BDD	Boron-Doped Diamond
CBC	Complete blood count
CFU	Colony-forming unit
CLSI	Clinical and Laboratory Standards Institute
EDTA	ethylenediaminetetraacetic acid
*E. coli*	*Escherichia coli*
EIS	electrochemical impedance spectroscopy
GRAN	granulocytes
H&E	Hematoxylin and eosin
HCT	hematocrit
HGB	hemoglobin
IACUC	Institutional Animal Care and Use Committee
LB	Luria–Bertani
LSV	linear sweep voltammetry
LYM	lymphocytes
MCH	mean corpuscular hemoglobin
MCHC	mean corpuscular hemoglobin concentration
MCV	mean corpuscular volume
MON	monocytes
MPV	mean platelet volume
NIH	National Institutes of Health
OEP	oxygen evolution overpotential
*P. aeruginosa*	*Pseudomonas aeruginosa*
PBS	phosphate-buffered saline
PCT	plateletcrit
PFA	paraformaldehyde
PDW	platelet distribution width
PEM	proton exchange membrane
PLT	platelet
RBC	Red blood cell
RDW	red cell distribution width
RHE	reversible hydrogen electrode
ROS	Reactive Oxygen Species
*S. aureus*	*Staphylococcus aureus*
SEM	scanning electron microscopy
SPF	specific-pathogen-free
UV	ultraviolet
WBC	White blood cell
XRD	X-ray diffraction
